# Gene expression dynamics in *Bacillus cereus* and *Bacillus subtilis* treated with *Thymus vulgaris* and *Origanum vulgare* subsp. *hirtum* essential oils

**DOI:** 10.3389/fmicb.2025.1643608

**Published:** 2025-08-22

**Authors:** Fabrizio Anniballi, Chiara Purgatorio, Annalisa Serio, Concetta Scalfaro, Silvia Taglieri, Antonello Paparella

**Affiliations:** ^1^National Reference Centre for Botulism, Department of Food Safety, Nutrition and Veterinary Public Health, Istituto Superiore di Sanità, Rome, Italy; ^2^Department of Bioscience and Technology for Food, Agriculture and Environment, University of Teramo, Teramo, Italy

**Keywords:** *Bacillus cereus*, *Bacillus subtilis*, *Thymus vulgaris*, *Origanum vulgare* subsp. *hirtum*, essential oils, gene expression *Bacillus cereus*, gene expression *Bacillus subtilis*

## Abstract

Essential oils (EOs) hold significant potential as antimicrobials in food, due to their high concentration of active phenolic compounds. These compounds can target bacterial cells through various mechanisms, such as membrane disruption, *quorum sensing* inhibition, and interference in virulence factors, affecting microorganisms at a genomic level. *Bacillus cereus* and *Bacillus subtilis* are key foodborne bacteria that could be managed using these natural preservatives. The present study investigated the effects of stress induced by applying *Thymus vulgaris* and *Origanum vulgare* subsp. *hirtum* EOs on genetic modifications in *B. cereus* 11 and *B. subtilis* 58C strains isolated from shelf-stable gnocchi, through their gene expression analysis by quantitative real-time RT-PCR. Sublethal EO concentrations were tested, at increasing time intervals (6, 12, 18, 24, and 48 h). Most of the genes were downregulated at 6 h, indicating that the stressful situation prolonged the lag phase. Only *spo0A* for both *B. cereus* and *B. subtilis*, and *pbpF* and *sigB* for *B. subtilis* were upregulated after 6 h, suggesting an attempt to restore cellular communication and repair membrane damage. The *pbpF* gene was the most significant in the stress response of *B. subtilis*. Conversely, *B. cereus* responded through different mechanisms, primarily driven by the *plcR* and *nheB* genes, illustrating the role of virulence mechanisms in its stress response. In both strains, the genes were generally more upregulated at a higher concentration of EO (0.58 mg/mL), which was more stimulating than at 0.29 mg/mL. Moreover, the two EOs elicited variable stress responses, which implies different cellular mechanisms and genes in the same microorganism. Therefore, the outcomes of this study suggest that the action of the two EOs mainly influenced cell membrane integrity and *quorum sensing* mechanisms, with differences in the genes involved for the two species and the two EOs.

## 1 Introduction

*Bacillus cereus* and *Bacillus subtilis* are spore-forming, Gram-positive, rod-shaped bacteria that are commonly found in the environment, particularly in soil, sediments, dust, plants, and foods. These two species are of interest for a variety of foods, particularly starchy and potato-based products, where they are frequently detected (Del Torre et al., 2001; Purgatorio et al., 2024). Due to their ability to form spores, *Bacillus* spp. pose a concern for food products, even if thermally treated. Indeed, sporulation confers resistance to various stressful situations, including those induced by heat, high hydrostatic pressure, acids, antibiotics, or other antimicrobials (Stenfors Arnesen et al., 2008; Chaves López et al., 2009; Jin et al., 2021).

*B. cereus* is well recognised as a spoilage agent and a pathogen in foods. This microorganism is responsible for two types of foodborne diseases: the emetic syndrome, which is caused by consuming foods contaminated with cereulide, and diarrhoea, along with abdominal pain, due to toxins produced by enteropathogenic strains in the small intestine. For the latter disease, the infectious dose is estimated between 10^5^ and 10^8^ CFU/g viable cells or spores (Stenfors Arnesen et al., 2008). *B. cereus-*associated gastroenteric diseases are mostly mild and self-limiting; however, some fatal cases have been reported (Hoffmaster et al., 2008).

*B. subtilis* is a particularly competitive microorganism that adapts well to different environmental conditions due to its ease of genetic modification. In the food industry, *B. subtilis* can be used as a starter for fermentation (Kovács, 2019) and for other technological applications, such as chitosan production (Sini et al., 2007) or the antifungal activity of its volatile compounds (Chaves López et al., 2015). Additionally, while possessing probiotic and functional properties, *B. subtilis* strains can also lead to food spoilage, characterised by red discoloration, slime formation, and a sticky texture (Purgatorio et al., 2024).

Purgatorio et al. (2024) have recently investigated the occurrence of various species of *Bacillus* spp. in ambient gnocchi. *B. subtilis* and *B. cereus* were the two most frequently isolated species in the samples formulated without the organic acids traditionally used as preservatives. The removal of conventional additives in food formulations is becoming increasingly popular, and research is shifting towards the use of natural substitutes. This trend is connected to the rising phenomenon of antimicrobial resistance and the growing interest in clean label foods, to create alternative and eco-friendly products. Essential oils (EOs) are particularly appealing among these natural alternatives for their well-documented antimicrobial properties (D’Amato et al., 2018).

In fact, EOs can act against target cells through several mechanisms, including the destabilisation of cytoplasmic membrane phospholipids, leakage of cellular material, loss of ions, protein denaturation, interference with *quorum sensing* mechanisms, sporulation, and the expression of virulence factors (Purgatorio et al., 2022; Rossi et al., 2022). Therefore, the contact with EOs represents a stressful event for the cells, which attempt to adapt and survive by regulating the expression of a wide range of genes. The molecular targets that may be affected include those involved in *quorum sensing* and virulence (e.g., *plcR, nhe* genes) (Jin et al., 2021; Rutherford and Bassler, 2012). As previously demonstrated in *B. cereus*, applying sublethal concentrations of biopreservatives can hinder vital communication between bacteria, resulting in reduced expression of virulence factors (Jin et al., 2021). The alteration of communication within the bacterial population can also influence their biofilm production and the gene expression involved in its formation (e.g., *codY, sinR, spo0A*) (Lindbäck et al., 2012; Jin et al., 2021; Xu et al., 2017; Zhao et al., 2021). Other genes that may be affected by biopreservative-induced stress include those involved in cellular metabolism and growth control (Jin et al., 2021) and the maintenance of membrane integrity (e.g., *pbpF*) (Chowdhury et al., 2021).

*Thymus vulgaris* and *Origanum vulgare* subsp. *hirtum* EOs have been extensively studied for their antimicrobial properties, which are associated with the high level of phenolic compounds, such as thymol, carvacrol, *p*-cymene, γ-terpinene, and linalool (D’Amato et al., 2024; Kosakowska et al., 2024; Pellegrini et al., 2018; Tardugno et al., 2022).

This study aims to evaluate the impact of treatments with sublethal concentrations of *Thymus vulgaris* and *Origanum vulgare* subsp. *hirtum* EOs on the expression of the genes involved in the stress response in *B. cereus* and *B. subtilis*.

## 2 Materials and methods

### 2.1 Bacterial strains and culture conditions

*B. cereus* strain 11 and *B. subtilis* strain 58C, isolated and characterized in a previous study (Purgatorio et al., 2024), were used in the design of experiments. *B. cereus* strain 11 was isolated from ambient gnocchi prepared without preservatives, packed in a modified atmosphere (MAP) and stored at room temperature (~25°C) for 5 days. The pathogenicity of this strain was underlined by the fact that it encodes the *cesC* gene, which is involved in the biosynthesis of the cereulide toxin, responsible for *B. cereus* emetic syndrome. *B. subtilis* strain 58C was isolated from ambient gnocchi containing lactic acid as a preservative, packed in MAP and stored under thermal abuse (30°C) for 7 days. The details of the isolation, identification, and molecular characterisation of the strains are reported in the previous study (Purgatorio et al., 2024). The strains were stored at −80°C in cryovials containing Brain Heart Infusion broth (BHI) (Oxoid, UK) and 20% (w/v) glycerol.

Each strain was streaked onto BHI agar and incubated at 37°C for 24–48 h. A single colony from each strain was then inoculated into BHI broth and incubated at 37°C for 18 h to obtain a fresh working culture.

### 2.2 Essential oils

Commercial and food-grade *Thymus vulgaris* thymol chemotype EO was kindly provided by Flora S.r.l. (Pisa, Italy), while *Origanum vulgare* subsp. *hirtum* carvacrol chemotype EO was kindly supplied by Exentiae S.r.l. Soc. Agricola (Catania, Italy). EOs were prepared at a concentration of 36.0 mg/mL, by adding PBS (Phosphate Buffer Saline) and Tween 80 (10.0 μL/mL). Homogeneous emulsions, obtained through vortexing, were subsequently sterilised using a 0.22 μm polytetrafluoroethylene (PTFE) Minisart syringe filter (Sartorius, Göttingen, Germany).

### 2.3 Primers design and SYBR green real-time PCR optimisation

The genes used in this study, presented in Table 1, were retrieved from the literature (Fouet et al., 2000; Hecker et al., 2007; Jin et al., 2021). Their functions are summarised in Table 2. The nucleotide sequence of each gene was extracted from publicly available reference genomes at accession numbers CP020383 and CP34551. These sequences were aligned with those of *B. cereus* strain 11 and *B. subtilis* strain 58C using the Clustal Omega algorithm.^1^ The consensus sequences obtained for each gene harboured by both *B. cereus* and *B. subtilis* generated by the alignment study were used to design primers through Beacon Design version 7.91 (Premier Biosoft International, USA).

**Table 1:** Target genes and primer set.

Organism	Gene	Primer	Primer sequence (5′ → 3′)	Product size (bp)
*B. cereus* strain 11	16S	16S_bc_f	CGGATAATATTTTGAACTGCATA	100
		16S_bc_r	CCGTTACCTCACCAACTA	
	*sigB*	sigB_bc_f	TAGCGATATGCAATAGAATAGA	131
		sigB_bc_r	CAACCTACGAATCTTACTAAAG	
	*sinR*	sinR_bc_f	ATCGCAGCAGTTCTACAA	81
		sinR_bc_r	CCATTCGGAGTCTAGGTTAG	
	*pbpF*	pbpF_bc_f	GTGGCTATATGATGGATGAA	113
		pbpF_bc_r	TGGCACCAAGTCTCTATT	
	*plcR*	plcR_bc_f	AGTGAGCCGAATTGAATC	89
		plcR_bc_r	AATGGATAATGGGAACTTGTA	
	*nheB*	nheB_bc_f	CTGTCGCAATCACTACTG	76
		nheB_bc_r	ATTATTATCGGCTCATCTGTT	
	*spo0A*	spo0A_bc_f	TCGTCCTTATTCGGTTAT	100
		spo0A_bc_r	CCTTATGTTCAAGTCTCAG	
*B. subtilis* strain 58C	16S	16S_bs_f	TCGGAGAGTTTGATCCTG	140
		16S_bs_r	CAGTCTTACAGGCAGGTTA	
	*sigB*	sigB_bs_f	GAGAAACAAATCATAGACCTTACG	78
		sigB_bs_r	TTGAGATATACCGAGAATGTCC	
	*codY*	codY_bs_f	TCAATTCAATGACGATGACTTA	106
		codY_bs_r	GCTTCTTGCTTCCTCTTC	
	*sinR*	sinR_bs_f	AAAGTCTCCGCTGTTCTG	83
		sinR_bs_r	CTATCTAATTGACCATCGTATTCG	
	*pbpF*	pbpF_bs_f	GCTACATTGACCTTGTGAT	77
		pbpF_bs_r	GTATCCGCCTTGAAGAAG	
	*spo0A*	spo0A_bs_f	ATATAGAAGGACAGGAAGA	105
		spo0A_bs_r	TATCTAATACGAGCACATC	

**Table 2:** Functions of target genes.

Gene name	Abbreviation	Function
RNA polymerase sigma factor SigB	*sigB*	General stress response (Hecker et al., 2007).
Class A penicillin-binding protein 2C	*pbpF*	Membrane integrity, peptidoglycan biosynthesis (Hadjilouka et al., 2017; Yoshikazu et al., 2009).
Pleiotropic transcriptional regulator	*codY*	Promotion of mobility, flagella expression, biofilm inhibition (Jin et al., 2021; Lindbäck et al., 2012).
Transcriptional regulator (Xre family) of post-exponential-phase responses genes	*sinR*	Control of biofilm formation, promotion of mobility (Xu et al., 2017).
Phosphorelay response regulator	*spo0A*	Control of biofilm formation, initiation of sporulation (Xu et al., 2017).
Pleiotropic regulator of extracellular virulence factor	*plcR*	Control of extracellular virulence, *quorum sensing* (Agaisse et al., 1999; Jin et al., 2021; Rutherford and Bassler, 2012; Yehuda et al., 2018)
Non-haemolytic enterotoxin regulator	*nheB*	Control of extracellular virulence (Hansen and Hendriksen, 2001)

The specificity of each primer was assessed through a basic local alignment search on BLASTn.^2^ The selectivity study (inclusivity and exclusivity) was performed *in silico* by running the freely available PCR amplification tool at the website http://insilico.ehu.es/, using the most permissive PCR conditions, against all available *Bacillus* species. Additional selectivity studies were conducted, testing each primer couple against the following strains: *Bacillus cereus* ATTC 11778, *Bacillus cereus* ATCC 27884, *Bacillus coagulans* ATCC 7050, *Bacillus subtilis* ATCC 6633, *Citrobacter freundii* ATCC 8090, *Clostridium botulinum* ATCC19397, *Clostridium butyricum* ATCC19398, *Enterococcus faecalis* ATCC 29212, *Escherichia coli* ATCC 25922, *Listeria innocua* ATCC 33090, *Listeria monocytogenes* ATCC 7644, *Pseudomonas aeruginosa* ATCC 9027, *Salmonella enterica* ser. Enteritidis ATCC 13076, *Staphylococcus aureus* ATCC 13565, *Streptococcus thermophilus* ATCC 19258 and *Rhodococcus equi* ATCC 6939, along with 13 wild strains isolated from ambient gnocchi (6 *B. subtilis,* 6 *B. cereus*, and 1 *B. atrophaeus*). All real-time PCR runs assessing selectivity were conducted in a Rotor-Gene Q (Qiagen, Hilden, Germany) real-time PCR machine using QuantiNova SYBR Green master mix (Qiagen, Hilden, Germany – cat. No 208056) according to the conditions suggested by the manufacturer. Inclusivity was defined as the ability of the PCR method to detect the target analyte from a wide range of strains. Exclusivity was defined as the lack of interference from a relevant range of nontarget strains in the PCR methods (Malorny et al., 2003).

Further optimisation involved identifying the optimal annealing temperature for each primer couple (range 55°C–59°C) and the best primers concentration (400, 600, 700, and 800 nM). Lastly, the dynamic range of linearity and amplification efficiency was assessed for each primer couple, testing a known quantity of DNA extracted from *B. cereus* strain 11 and *B. subtilis* strain 58C in triplicate. To evaluate amplification efficiency, a standard curve was plotted with the log of the number of DNA copies (x-axis) extracted from *B. cereus* strain 11 and *B. subtilis* strain 58C against the threshold cycle (Ct) for these copies (y-axis) (data not shown). The amplification efficiency (E) for the various targets was calculated using the following equation:

$$E=10^{-1/\text{slope}}-1$$

DNA copies were calculated using the following equation:

$$\text{Genomes}=\frac{\left(\mathrm{ng}\;\text{of}\;\mathrm{DNA}\;\text{spectrophotometrically measures}\times N\right)}{660\times \text{genome size}}$$

in which N represent Avogadro number (6,022 × 10^23^).

### 2.4 Treatment with *Thymus vulgaris* and *Origanum vulgare* subsp. *hirtum* essential oils

*B. cereus* strain 11 and *B. subtilis* strain 58C were treated with *T. vulgaris* and *O. vulgare* EOs using the broth microdilution method described by Clinical and Laboratory Standards Institute guidelines (CLSI, 2020). The EOs emulsions prepared at a concentration of 36.0 mg/mL were two-fold diluted in BHI broth in 2-mL Eppendorf tubes, to obtain concentrations ranging from 18.0 to 0.58 mg/mL. Cells from the 18 h culture were collected by centrifugation at 13,000 rpm (Eppendorf-Centrifuge 5415D, Hamburg, Germany) for 5 min and washed three times with PBS. The inoculum was standardised using a Jenway 6305 spectrophotometer at 5 × 10^5^ CFU/mL and tested in duplicate. Positive controls (BHI broth and inoculum) and negative controls (*T. vulgaris* or *O. vulgare* EOs and BHI broth) were also tested. Tubes were incubated at 37°C for 6, 12, 18, 24, and 48 h to assess the effect of EOs on *B. cereus* strain 11 and *B. subtilis* strain 58C at various exposure times. The lowest concentration of EOs that inhibited microbial growth after incubation at 37°C for 48 h was considered the *Minimum Inhibitory Concentration* (MIC). The analyses were carried out in triplicate.

### 2.5 RNA extraction

*B. cereus* strain 11 and *B. subtilis* strain 58C RNA were extracted from the tubes with EOs treatments. The concentrations to be tested were selected based on the MIC results at 48 h. Two sub-inhibitory concentrations were considered: 0.29 mg/mL (1/4 MIC) and 0.58 mg/mL (1/2 MIC) for both *Bacillus* species. The extractions were carried out by RNeasy Mini Kit (Qiagen, Germany) with some modifications. Briefly, 1 mL of each culture was chilled on ice for 30 min prior to extraction to limit metabolic and enzymatic activity. Cells were recovered by centrifugation at 13,000 rpm for 3 min and then washed four times with PBS. The pellet was resuspended in 500 μL of TE buffer, and 2 μL of lysozyme (20 mg/mL) was added. Samples were subsequently incubated at 37°C for 60 min to allow for cell rupture. Then, 700 μL of RLT buffer and 500 μL of 70% ethanol were added to the suspension, homogenising gently with the micropipette without vortexing. Then, 600 μL of the samples were transferred to a RNeasy Mini spin column and were centrifuged at 10,000 rpm for 1 min. The supernatant was discarded, and the process was repeated twice with the remaining sample. DNA digestion was performed by incubating the spin columns at 37°C for 30 min, in which 20 μL of DNase I solution and 140 μL of RDD buffer were deposited. After DNA digestion, 500 μL of buffer RPE was added to the spin columns and centrifuged at 10,000 rpm for 2 min. RNA was eluted in 100 μL of RNase-free water.

The RNA purity was assessed by a Nanodrop spectrometer (Thermo Fisher Scientific, United States), considering the A260/A280 ratio acceptable between 1.8 and 2.1. The RNA integrity was evaluated with the Agilent Technologies 2,100 Bioanalyzer (Agilent Technologies, Santa Clara, CA). The absence of DNA was confirmed by running 3 μL of each RNA sample as a template for real-time PCR, using the optimised protocols described above (no amplification demonstrated the absence of DNA traces).

### 2.6 Quantitative real-time RT-PCR SYBR green

RT-PCR SYBR Green reactions were conducted in Rotor-Gene Q (Qiagen, Hilden, Germany) using the QuantiNova SYBR Green RT-PCR kit (Qiagen, Hilden, Germany – cat. No 208154). Each assay was performed with 2 μL of template RNA, 10 μL of SYBR Green RT-PCR Master Mix, 0.2 μL of QuantiNova SYBR Green RT-Mix, variable volumes of each primer, according to the concentration selected during optimisation (see Table 3), along with RNase-free water to reach a total volume of 20 μL. The RT-PCR conditions consisted of an initial RT-step at 50°C for 10 min to allow cDNA production, followed by 40 cycles of PCR initial heat activation at 95°C for 2 min (2-step cycling) and denaturation at 95°C for 5 s and followed by combined annealing/extension for 10 s at the optimised temperature. No-RT and no-template controls were included in each run to check for contamination of reagents. Gene expression studies were carried out according to the MIQE guidelines.^3^

**Table 3:** PCR protocol and performance parameters.

Primer	Primer concentration (nM)	Annealing temperature (°C)	Melting temperature of PCR products (°C)	Linearity (orders of magnitude)	PCR efficiency (%)	*R* ^2^
16S_bc_f	800	59	80.80 ± 0.10	7	100.61	0.9998
16S_bc_r	800					
sigB_bc_f	600	59	76.25 ± 0.10	7	86.30	0.999
sigB_bc_r	600					
spo0A_bc_f	400	59	78.40 ± 0.15	7	75.77	0.9997
spo0A_bc_r	400					
sinR_bc_f	600	59	76.00 ± 0.25	7	88.67	0.9957
sinR_bc_r	600					
pbpF_bc_f	800	59	77.65 ± 0.15	7	94.40	0.9974
pbpF_bc_r	800					
plcR_bc_f	400	59	78.0 ± 0.10	7	89.51	0.9989
plcR_bc_r	400					
nheB_bc_f	400	59	78.55 ± 0.05	7	86.59	0.9989
nheB_bc_r	400					
16S_bs_f	400	59	84.85 ± 0.15	7	87.10	0.9966
16S_bs_r	400					
sigB_bs_f	800	59	74.45 ± 0.15	7	88.75	0.9965
sigB_bs_r	800					
codY_bs_f	600	59	77.75 ± 0.10	7	95.44	0.9987
codY_bs_r	600					
spo0A_bs_f	800	59	79.60 ± 0.10	7	84.19	0.9994
spo0A_bs_r	800					
sinR_bs_f	600	59	78.15 ± 0.15	7	87,10	0.9957
sinR_bs_r	600					
pbpF_bs_f	600	59	78.45 ± 0.20	7	96.60	0.9981
pbpF_bs_r	600					

### 2.7 Statistical analysis

All the real-time PCR experiments were conducted in triplicate. Arithmetic means and standard deviations of melting temperature (Tm) values were calculated to define positive results. A positive result was assigned to an assay that generated a Ct value at the expected Tm for the target gene. RT-qPCR runs were performed in triplicate using *16S rrn* as the reference gene. Normalised data were converted to relative expression as described by Pfaffl (2001) and log2-values (fold change) according to Kubista (2007) for further analysis with one-way ANOVA. *p* < 0.05 was considered significant. Data visualisation and statistical analysis were performed using Microsoft Excel and Prism 9.5.1 software (GraphPad, Boston, MA, USA).

## 3 Results

### 3.1 SYBR green real-time PCR protocols and their performance parameters

Each primer pair was designed to produce a specific signal for *B. cereus* strain 11 and *B. subtilis* strain 58C. Table 3 shows the PCR conditions optimised for each set of primers using the QuantiNova master mix in the Rotor-Gene Q thermal cycler. The melting temperature (Tm) of PCR products, obtained by running positive control DNAs, was used to distinguish between positive and negative results. The selectivity study provided 100% inclusivity and 100% exclusivity (data not shown).

As reported in Table 3, all primer sets produced a linear response over seven orders of magnitude with PCR efficiency ranging from 84.19 to 100.61%.

### 3.2 Treatment with *Thymus vulgaris* and *Origanum vulgare* subsp. *hirtum* essential oils

MIC values of *T. vulgaris* and *O. vulgare* EOs against *B. cereus* strain 11 and *B. subtilis* strain 58C after 24 h and 48 h of exposure are summarised in Table 4.

**Table 4:** MIC values after 24 h and 48 h of exposure to *T. vulgaris* and *O. vulgare* EOs.

Strain	*T. vulgaris* EO		*O. vulgare* EO	
	MIC 24 h	MIC 48 h	MIC 24 h	MIC 48 h
*B. cereus* strain 11	0.58	1.16	0.58	1.16
*B. subtilis* strain 58C	0.58	1.16	0.58	1.16

MIC values are expressed in mg/mL.

Based on the obtained MIC values, cultures of the mentioned strains exposed to sub-inhibitory concentrations of 0.29 mg/mL (1/4 MIC for 48 h) and 0.58 mg/mL (1/2 MIC for 48 h) were subjected to RNA extraction and gene expression analysis.

### 3.3 Effect of *Thymus vulgaris* and *Origanum vulgare* subsp. *hirtum* essential oils on gene expression

Results of relative gene expression are presented in Tables 5, 6, and Figures 1–8.

**Table 5:** Effect of *Thymus vulgaris* EO on *B. cereus* strain 11 and *B. subtilis* strain 58C at different exposure times.

Strain	Gene	EO concentration (mg/mL)	Relative gene expression at different times				
			6 h	12 h	18 h	24 h	48 h
*B. cereus* strain 11	*sigB*	0.58	1.05 ± 0.26	0.99 ± 0.27	**2.01 ± 0.23**	**0.32 ± 0.07**	0.00
		0.29	**0.26 ± 0.03**	**0.35 ± 0.04**	1.40 ± 0.25	**1.23 ± 0.26**	4.19 ± 3.62
	*sinR*	0.58	0.43 ± 0.05	**3.05 ± 0.09**	0.51 ± 0.01	**2.50 ± 0.15**	0.60 ± 0.08
		0.29	0.00	0.66 ± 0.09	**4.90 ± 1.09**	**7.60 ± 1.11**	1.58 ± 0.02
	*pbpF*	0.58	**0.66 ± 0.15**	**1.64 ± 0.42**	**2.01 ± 0.27**	1.29 ± 0.10	0.57 ± 0.19
		0.29	**0.39 ± 0.04**	**0.35 ± 0.04**	**1.78 ± 0.17**	6.49 ± 2.10	**3.41 ± 0.38**
	*plcR*	0.58	0.50 ± 0.05	4.26 ± 1.48	**5.61 ± 0.27**	**40.13 ± 8.77**	**5.38 ± 0.84**
		0.29	**0.04 ± 0.00**	**44.34 ± 7.75**	**10.74 ± 1.87**	**179.13 ± 24.34**	**12.73 ± 1.14**
	*nheB*	0.58	**0.02 ± 0.00**	**1.55 ± 0.15**	**2.85 ± 0.08**	**6.52 ± 1.27**	**2.80 ± 0.38**
		0.29	**0.06 ± 0.00**	0.56 ± 0.10	**13.78 ± 1.65**	**0.72 ± 0.03**	**2.97 ± 0.30**
	*spo0A*	0.58	1.19 ± 0.41	**5.08 ± 0.89**	0.00	0.00	1.25 ± 0.33
		0.29	**2.94 ± 0.28**	**11.24 ± 0.51**	0.00	0.00	**15.17 ± 2.73**
*B. subtilis* strain 58C	*sigB*	0.58	0.65 ± 0.07	**0.83 ± 0.03**	**0.34 ± 0.03**	**0.50 ± 0.02**	**4.55 ± 0.73**
		0.29	**6.43 ± 1.22**	0.00	0.00	**0.06 ± 0.01**	0.81 ± 0.05
	*sinR*	0.58	0.62 ± 0.14	1.17 ± 0.23	**0.49 ± 0.05**	0.95 ± 0.08	**8.74 ± 0.61**
		0.29	**0.48 ± 0.07**	0.00	0.00	**0.38 ± 0.02**	1.60 ± 0.39
	*pbpF*	0.58	1.38 ± 0.15	**0.19 ± 0.03**	**0.82 ± 0.07**	**0.24 ± 0.03**	1.65 ± 0.89
		0.29	**30.35 ± 5.06**	**204.45 ± 48.05**	0.00	**0.52 ± 0.07**	1.41 ± 0.71
	*codY*	0.58	**0.38 ± 0.02**	1.28 ± 0.25	1.10 ± 0.09	**0.51 ± 0.04**	1.97 ± 0.57
		0.29	**0.17 ± 0.03**	2.89 ± 1.89	**0.31 ± 0.02**	**0.25 ± 0.05**	0.86 ± 0.29
	*spo0A*	0.58	**0.21 ± 0.07**	1.74 ± 0.67	**0.59 ± 0.02**	**0.58 ± 0.04**	0.00
		0.29	**26.86 ± 3.58**	**16.66 ± 3.54**	**474.71 ± 10.48**	0.00	0.26 ± 0.06

Values reported in bold indicate a statistically significant (*p* < 0.05) difference between the gene of interest and control (control relative gene expression = 1.00).

**Table 6:** Effect of *Origanum vulgare* EO on *B. cereus* strain 11 and *B. subtilis* strain 58C at different exposure times.

Strain	Gene	EO concentration (mg/mL)	Relative gene expression at different times				
			6 h	12 h	18 h	24 h	48 h
*B. cereus* strain 11	*sigB*	0.58	**3.09 ± 0.42**	**3.55 ± 0.77**	4.17 ± 1.57	**0.02 ± 0.00**	6.03. ± 2.66
		0.29	0.00	1.60 ± 0.04	**3.99 ± 0.45**	1.78 ± 0.60	**17.08. ± 2.20**
	*sinR*	0.58	0.15 ± 0.26	0.23 ± 0.03	0.79 ± 0.02	**2.49 ± 0.36**	**0.30 ± 0.09**
		0.29	0.00	0.22 ± 0.03	0.86 ± 0.06	1.68 ± 0.58	0.85 ± 0.09
	*pbpF*	0.58	**7.19 ± 1.71**	1.04 ± 0.19	1.96 ± 0.09	**42.65 ± 0.99**	**8.93 ± 2.17**
		0.29	0.00	2.55 ± 0.75	0.00	**0.39 ± 0.11**	**1.99 ± 0.19**
	*plcR*	0.58	0.53 ± 0.13	**2.72 ± 0.17**	**4.72 ± 0.77**	**0.04 ± 0.00**	9.32 ± 3.68
		0.29	0.00	**1.09 ± 0.39**	1.85 ± 0.46	2.11 ± 0.54	**7.68 ± 1.87**
	*nheB*	0.58	**0.32 ± 0.03**	**18.37 ± 3.87**	3.39 ± 0.49	**6.19 ± 1.06**	4.43 ± 1.60
		0.29	**0.05 ± 0.00**	**2.04 ± 0.24**	**3.73 ± 0.20**	**0.36 ± 0.10**	**8.67 ± 1.56**
	*spo0A*	0.58	**2.97 ± 0.14**	**1.43 ± 0.20**	0.18 ± 0.07	**24.57 ± 0.48**	**6.47 ± 1.35**
		0.29	**3.08 ± 0.57**	**63.16 ± 15.35**	0.41 ± 0.09	**0.45 ± 0.11**	**1.08 ± 0.14**
*B. subtilis* strain 58C	*sigB*	0.58	**3.03 ± 0.23**	**3.05 ± 0.21**	2.05 ± 0.62	**1.66 ± 0.35**	**1.97 ± 0.27**
		0.29	**0.10 ± 0.02**	**3.56 ± 0.80**	0.87 ± 0.17	**9.03 ± 0.52**	**2.39 ± 0.15**
	*sinR*	0.58	**2.62 ± 0.31**	**2.67 ± 0.39**	3.41 ± 0.92	**11.06 ± 0.89**	**4.83 ± 1.29**
		0.29	**0.14 ± 0.03**	1.70 ± 0.57	1.05 ± 0.06	**41.79 ± 11.13**	**7.20 ± 0.07**
	*pbpF*	0.58	0.00	**2.74 ± 0.52**	**5.03 ± 1.10**	2.65 ± 0.78	**3.86 ± 0.61**
		0.29	1.35 ± 0.35	**4.88 ± 1.23**	**8.13 ± 2.08**	**7.42 ± 0.46**	**2.11 ± 0.03**
	*codY*	0.58	1.26 ± 0.18	**9.54 ± 1.31**	**12.65 ± 4.45**	**2.65 ± 0.19**	6.59 ± 2.71
		0.29	**0.03 ± 0.01**	**10.26 ± 2.37**	**12.35 ± 1.82**	1.35 ± 0.08	**6.11 ± 0.87**
	*spo0A*	0.58	0.00	**0.25 ± 0.04**	**3.82 ± 0.97**	**9.32 ± 0.74**	0.07 ± 0.03
		0.29	0.00	0.71 ± 0.20	**16.03 ± 5.12**	**13.30 ± 0.09**	**0.19 ± 0.00**

Values reported in bold indicate a statistically significant (*p* < 0.05) difference between the gene of interest and control (control relative gene expression = 1.00).

**Figure 1:**
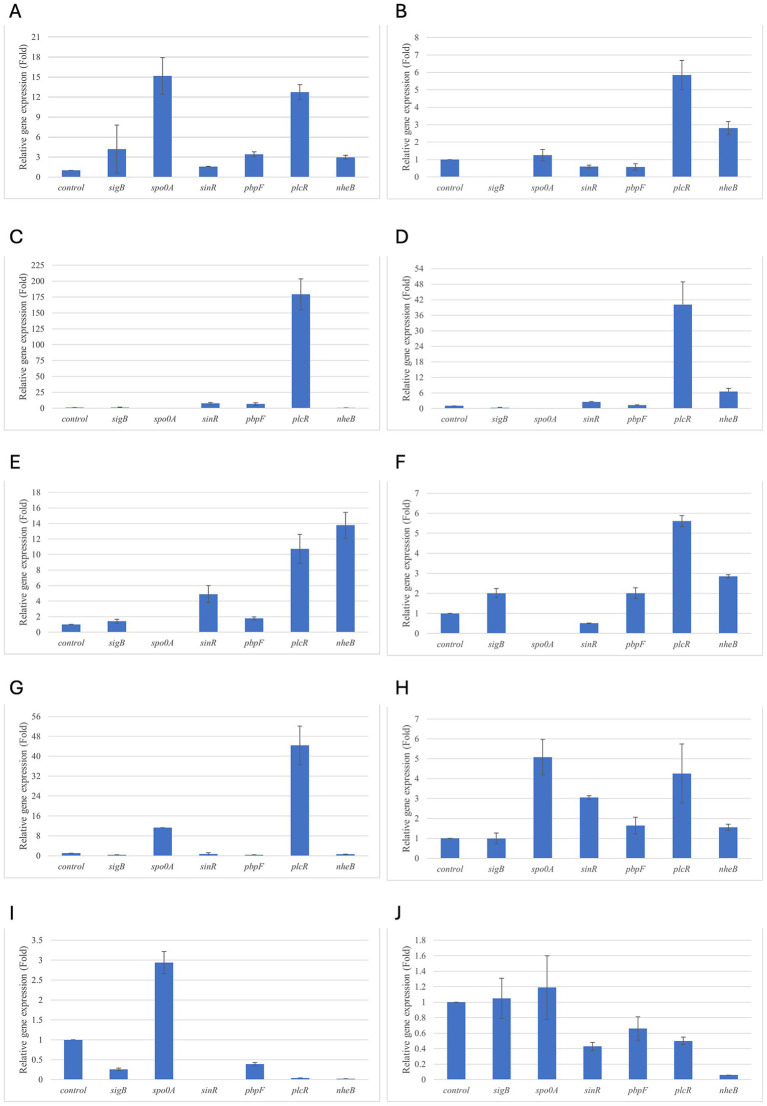
Relative gene expression of *B. cereus* strain 11 at various exposure durations to *Thymus vulgaris* EO. **(A)** 0.58 mg/mL for 48 h, **(B)** 0.29 mg/mL for 48 h, **(C)** 0.58 mg/mL for 24 h, **(D)** 0.29 mg/mL for 24 h, **(E)** 0.58 mg/mL for 18 h, **(F)** 0.29 mg/mL for 18 h, **(G)** 0.58 mg/mL for 12 h, **(H)** 0.29 mg/mL for 12 h, **(I)** 0.58 mg/mL for 6 h, **(J)** 0.29 mg/mL for 6 h. Bars depict the mean of three replicates, with error bars showing standard deviation.

**Figure 2:**
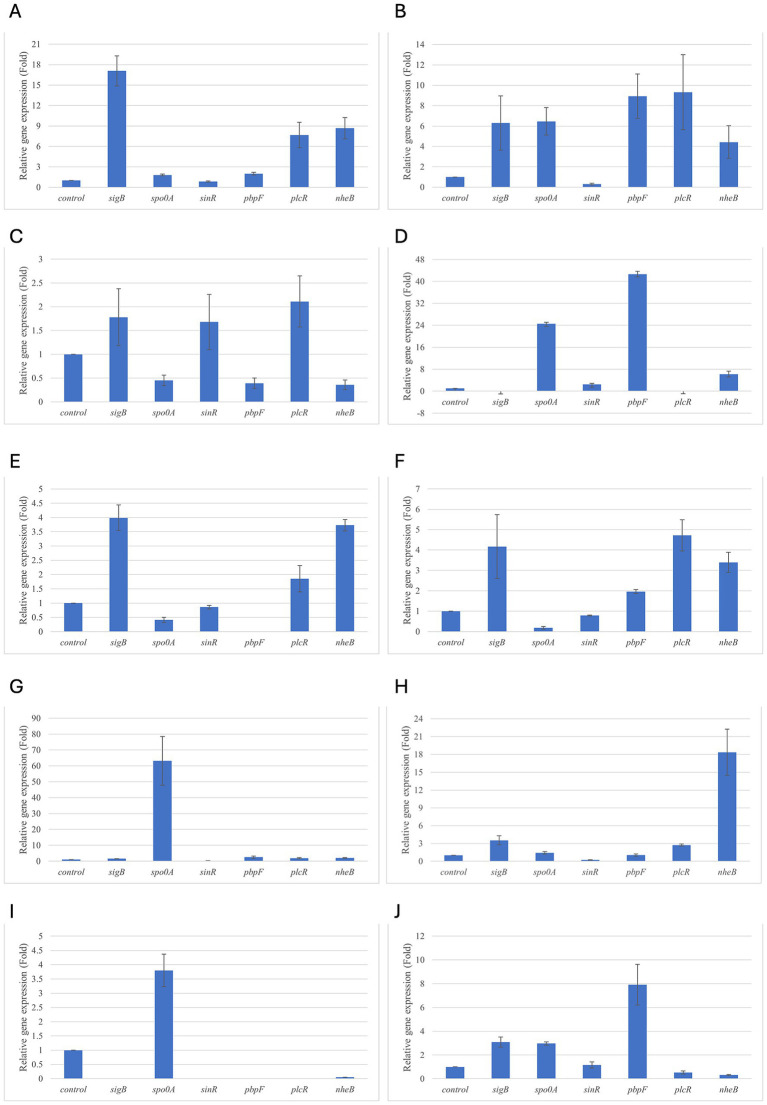
Relative gene expression of *B. cereus* strain 11 at various exposure durations to *Origanum vulgare* EO. **(A,B)** 48 h, **(C,D)** 24 h, **(E,F)** 18 h, **(G,H)** 12 h, **(I,J)** 6 h. Bars depict the mean of three replicates, with error bars showing standard deviation.

**Figure 3:**
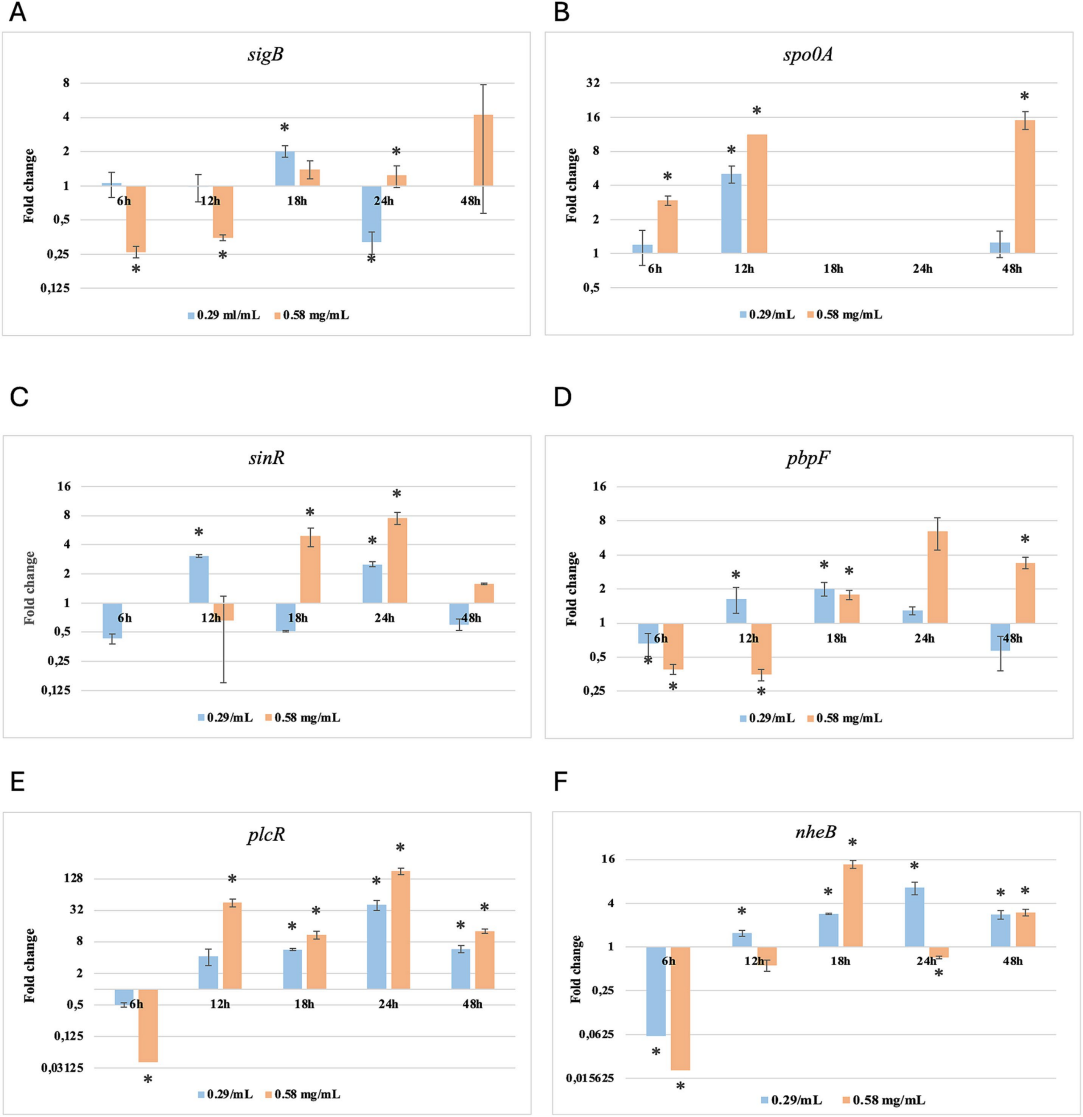
Log2 fold change of *B. cereus* strain 11 at various exposure durations to *Thymus vulgaris* EO. Bars depict the mean of three replicates, with error bars showing standard deviation. Asterisks (*) indicate significant differences between treatments and control (*p* < 0.05).

**Figure 4:**
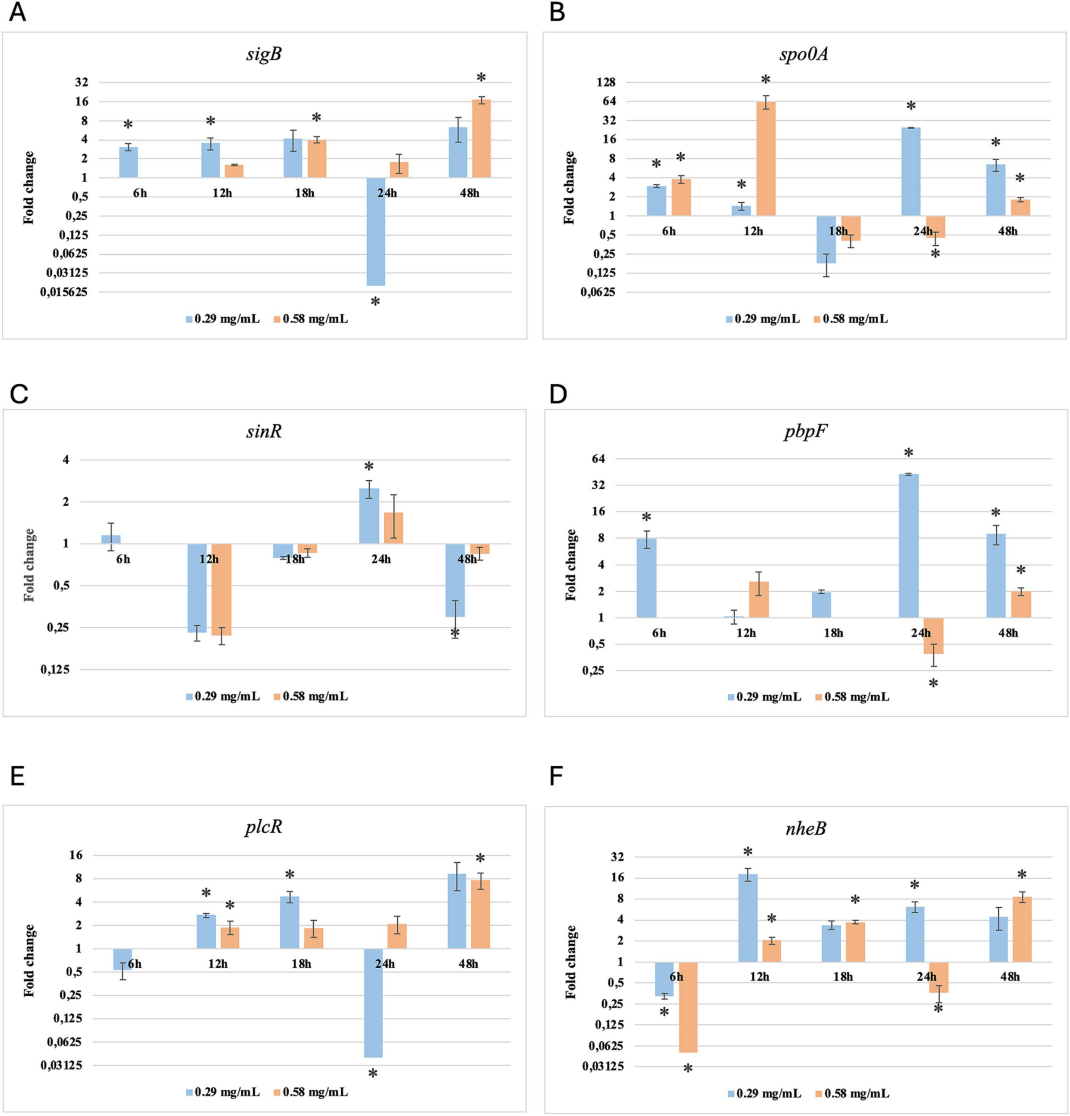
Log2 fold change of *B. cereus* strain 11 at various exposure durations to *Origanum vulgare* EO. Bars depict the mean of three replicates, with error bars showing standard deviation. Asterisks (*) indicate significant differences between treatments and control (*p* < 0.05).

**Figure 5:**
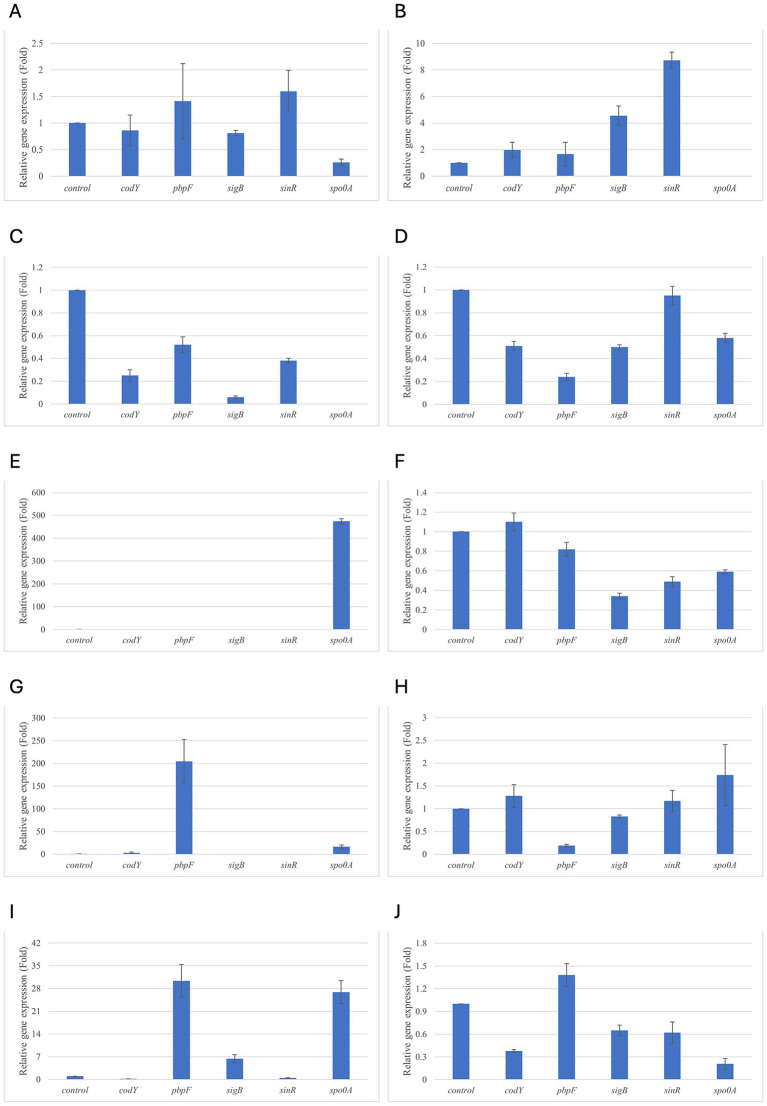
Relative gene expression of *B. subtilis* strain 58C at various exposure durations to *Thymus vulgaris* EO. **(A)** 0.58 mg/mL for 48 h, **(B)** 0.29 mg/mL for 48 h, **(C)** 0.58 mg/mL for 24 h, **(D)** 0.29 mg/mL for 24 h, **(E)** 0.58 mg/mL for 18 h, **(F)** 0.29 mg/mL for 18 h, **(G)** 0.58 mg/mL for 12 h, **(H)** 0.29 mg/mL for 12 h, **(I)** 0.58 mg/mL for 6 h, **(J)** 0.29 mg/mL for 6 h. Bars depict the mean of three replicates, with error bars showing standard deviation.

**Figure 6:**
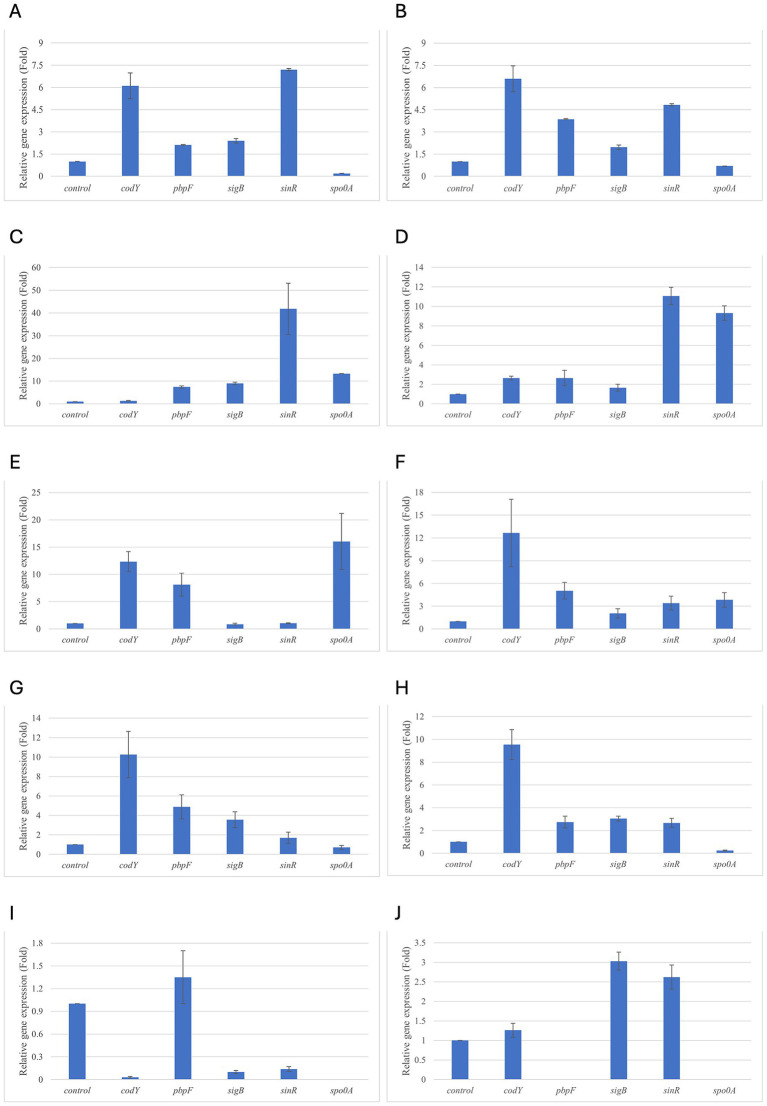
Relative gene expression of *B. subtilis* strain 58C at various exposure durations to *Origanum vulgare* EO. **(A)** 0.58 mg/mL for 48 h, **(B)** 0.29 mg/mL for 48 h, **(C)** 0.58 mg/mL for 24 h, **(D)** 0.29 mg/mL for 24 h, **(E)** 0.58 mg/mL for 18 h, **(F)** 0.29 mg/mL for 18 h, **(G)** 0.58 mg/mL for 12 h, **(H)** 0.29 mg/mL for 12 h, **(I)** 0.58 mg/mL for 6 h, **(J)** 0.29 mg/mL for 6 h. Bars depict the mean of three replicates, with error bars showing standard deviation.

**Figure 7:**
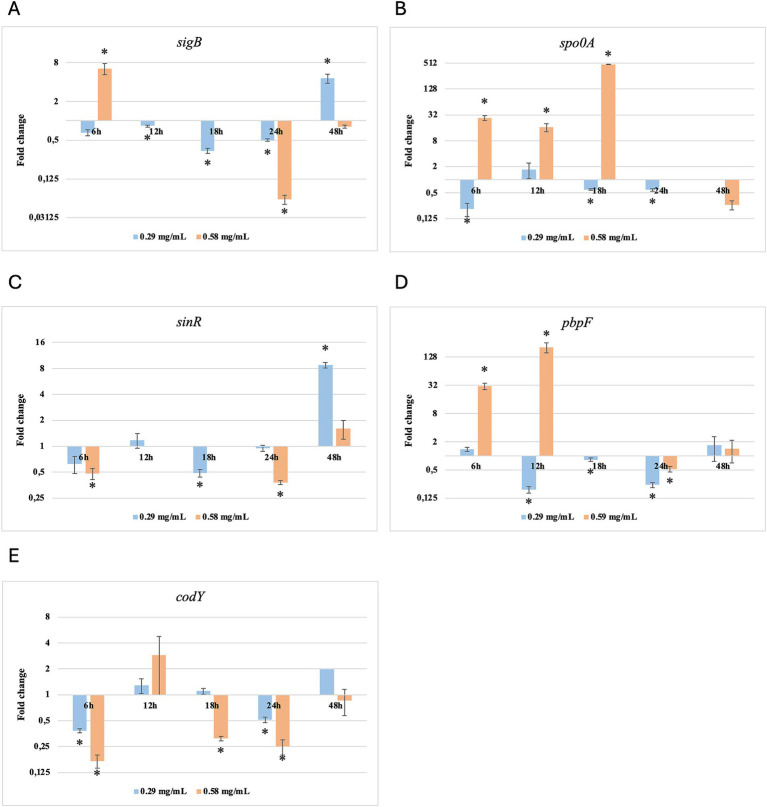
Log2 fold change of *B. subtilis* strain 58C at various exposure durations to *Thymus vulgaris* EO. Bars depict the mean of three replicates, with error bars showing standard deviation. Asterisks (*) indicate significant differences between treatments and control (*p* < 0.05).

**Figure 8:**
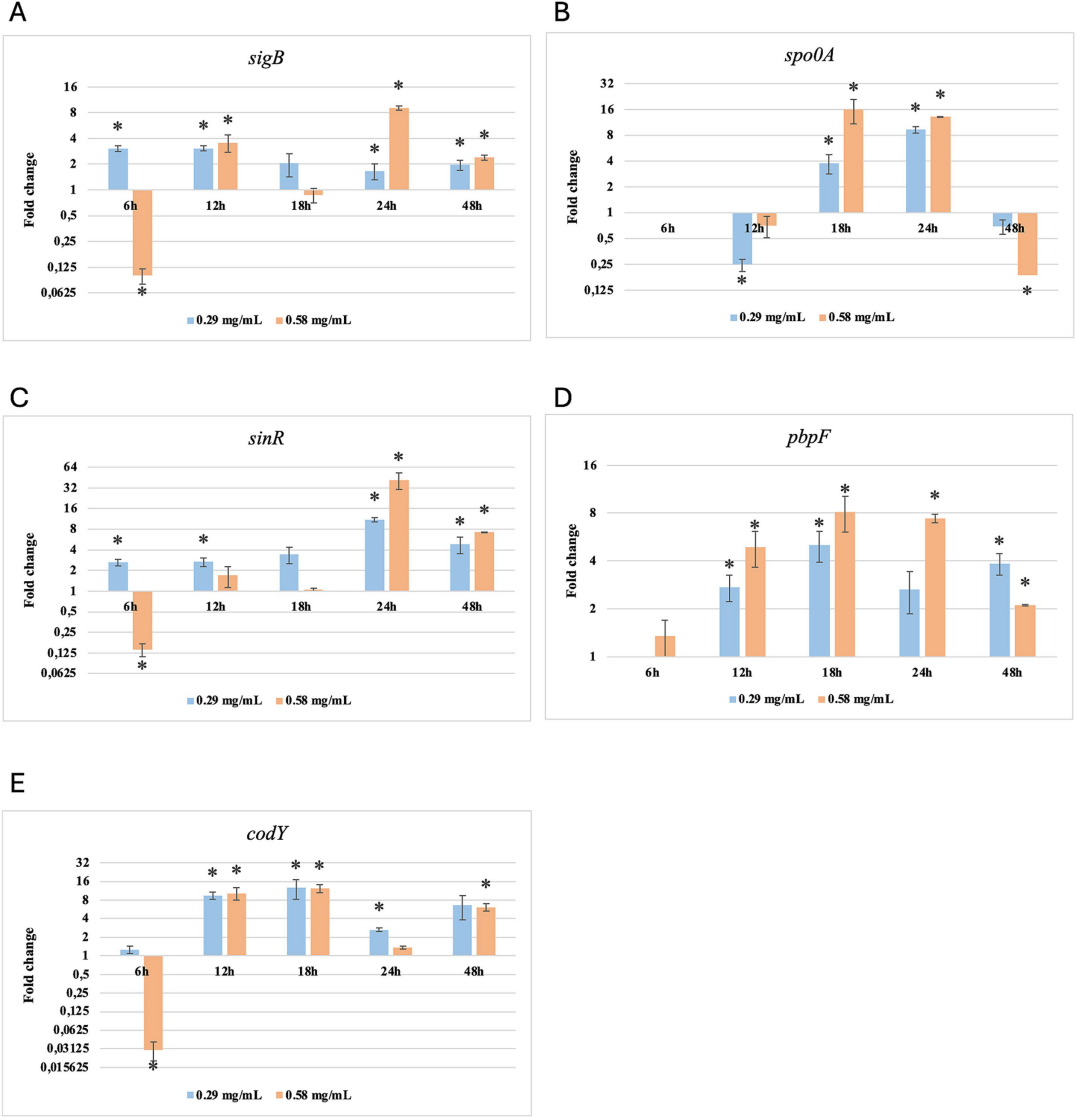
Log2 fold change of *B. subtilis* strain 58C at different exposure times to *Origanum vulgare* EO. Bars depict the mean of three replicates, with error bars showing standard deviation. Asterisks (*) indicate significant differences between treatments and control (*p* < 0.05).

After 6 h of exposure to both EOs, most of the genes in the two tested strains exhibited a downregulation, with some exceptions in which an upregulation was observed (*T. vulgaris*: *B. cereus spo0A; B. subtilis sigB, spo0A, pbpF; O. vulgare*: *B. cereus sigB, spo0A, pbpF; B. subtilis: sigB, sinR*) (Tables 5, 6; Figures 3B, 4A,B,D, 7A,B,D, 8A,C). The sigma factor B (*sigB*) was the most significantly upregulated gene (*p* < 0.05) at 6 h.

#### 3.3.1 *Bacillus cereus*: gene expression and comparison of exposure times

For *B. cereus*, after 12 h of exposure, almost all genes exhibited increased regulation levels compared to those seen at 6 h, likely because the strains began to organise their cellular functions to counteract the antimicrobial activity exerted by the EOs (Figures 1,2G,H). At this time, the genes that primarily increased their expression in *B. cereus* were *plcR* and *spo0A* for both EOs. For *O. vulgare* EO, *nheB* was significantly overexpressed at 12 h, while an evident upregulation began at 18 h for *T. vulgaris*. Generally, there was an upregulation at 18 h, but no high peaks were seen for 12 h (Figures 1,2E,F). This is likely because the cells, at the start of their stationary phase, had already implemented their stress response, thus maintaining these functions. Expression values exceeding 10.00 were observed for *plcR* and *nheB* (treatment with *T. vulgaris*), highlighting how the reaction to stressful situations involves pathogenicity factors of the microorganism. This trend was also evident at 24 h and 48 h, when genes associated with virulence continued to be overexpressed (Figures 1,2A–D). The highest values were recorded at 24 h for *plcR* (between approximately 40.00 and 180.00, *T. vulgaris* treatment), at which point the gene reached its peak level. Unexpectedly, *spo0A* subjected to *Thymus vulgaris* at 18 h and 24 h showed no expression levels.

*O. vulgare* treatment also influenced other genes at 24 h and 48 h, particularly *sigB*, *pbpF,* and *spo0A*, which were involved to a lesser extent in the response to stress caused by *T. vulgaris*. In particular, *pbpF* was significantly upregulated to values higher than 42.00 after *O. vulgare* EO exposure. This difference indicates that the cells could respond variably to the different EOs, because of their composition of active molecules. The *sinR* gene, for both EOs, was only modestly upregulated at 18–24 h, while it was downregulated at the other analysis times.

While for the most significant part of the genes there was no evident difference between the expression at the two concentrations tested, for other genes (*plcR, spo0A, sinR*) *T. vulgaris* EO determined a more significant overexpression at the concentration of 0.58 mg/mL, as indicated by the fold change (Figures 3B,C,E). In contrast, *O. vulgare* EO was more stimulating at 0.29 mg/mL for the other genes, particularly *pbpF* (Figure 4D).

#### 3.3.2 *Bacillus subtilis*: gene expression and comparison of exposure times

For *B. subtilis*, expression at 6 h was limited, as for *B. cereus*. The *sigB* gene was among the most upregulated initially, but its expression decreased during exposure to *T. vulgaris* EO, while it continued, with a peak at 24 h, during exposure to *O. vulgare* EO (Figures 7,8A). For *B. cereus*, gene expressions were generally higher at 12 h, when cells began to activate their defense mechanisms (Figures 5,6G,H). The *pbpF* gene was significantly expressed for both EOs, particularly for *T. vulgaris*, which peaked at approximately 205.00 at 12 h, following a high expression level at 6 h (about 30.35). This indicates that the *pbpF* gene is significantly involved in the gene expression of *B. subtilis*, especially during the first hours of exposure to *T. vulgaris* (Figure 7D). The same gene remained more consistently expressed up to 48 h for *O. vulgare* (Figure 8D). The *codY* gene was overexpressed at 12 h for both EOs, to a greater extent for *O. vulgare* (values around 10.00) (Figures 7,8E). The *spo0A* gene, instead, showed a significant role in the first hours of exposure only for *T. vulgaris*, while it was downregulated for *O. vulgare*. However, at 18 h, there was upregulation for both EOs, lower for *O. vulgare* (around 16.00 at a concentration of 0.58 mg/mL) and higher for *T. vulgaris*, with a peak of almost 475.00 at 0.58 mg/mL (Figures 7,8B).

At 18, 24, and 48 h, almost all the genes in *B. subtilis* treated with *T. vulgaris* EO showed significant downregulation, or slight non-significant upregulation (Figures 5A–F). This indicates that the active compounds in this EO trigger a response from the microorganism particularly in the early stages of exposure, especially at the highest concentration tested, when the stress was greater. Conversely, after exposure to *O. vulgare* for 18, 24, or 48 h, the level of overexpression remained relatively constant, and almost always significant for all tested genes (Figures 6A–F). This indicates that the molecules present in the *O. vulgare* EO act continuously, prompting the cell to implement numerous cellular functions. Furthermore, for this EO, the expression was similar for the two concentrations tested, with some exceptions where the higher concentration was more stimulating, particularly *sinR* at 24 h (0.29 mg/mL: about 11.00; 0.58 mg/mL: about 42.00).

## 4 Discussion

The multiple properties of EOs have been exploited since ancient times. Among EOs, those from the *Thymus* and *Origanum* genera are now widely used as herbal teas, tonics, carminatives, antitussives, and antiseptics. The species *T. vulgaris* and *O. vulgare* subsp. *hirtum* exhibit antibacterial and antifungal properties primarily due to their phenolic compounds content. It has been proposed that the minor components may be essential for the antimicrobial activity of EOs, because of their ability to establish synergistic effects (Gutierrez et al., 2008; Tardugno et al., 2022). Based on the profiles of aromatic compounds, *T. vulgaris* and *O. vulgare* have been classified into various chemotypes (Raal et al., 2024). In this study, the effect of two common chemotypes, *T. vulgaris* chemotype thymol and *O. vulgare* chemotype carvacrol, on the transcription of genes involved in the stress response in *Bacillus* spp. has been evaluated.

The aromatic compounds of EOs are “generally recognised as safe” (GRAS) by health food authorities (FDA (Food and Drug Administration), 2009). Due to their antimicrobial activity, they may represent promising alternatives to food preservatives and even antibiotics (Prakash et al., 2024). However, their sensory impact and the elevated cost may influence their practical application in the food industry. For this reason, the use of sublethal concentrations of EOs could be of great interest. This study aims to evaluate how *Bacillus* spp. strains respond at the genetic level to concentrations of EOs that are lower than the inhibitory dose. The genes *sigB*, *sinR*, *pbpF*, *spo0A, plcR*, and *nheB* were monitored in *B. cereus* strain 11, while *sigB*, *sinR*, *pbpF*, *spo0A*, and *codY* were observed in *B. subtilis* strain 58C. The effects of sub-inhibitory concentrations of EOs on these two strains were evaluated after 6, 12, 18, 24, and 48 h of exposure.

At the beginning of exposure (6 h), most of the tested genes were downregulated. Similarly, other authors have noted a delay in the lag phase in strains subjected to sub-inhibitory concentrations of EOs (Burt, 2004; Mazzarrino et al., 2015). During this period, cells organise their functions to respond to the stress they experience. At 6 h, one of the most upregulated genes was sigma factor B (*sigB*). It is a general transcription factor activated by various cellular stresses, including the pressure experienced upon entering the stationary growth phase. Harms et al. (2024) describe *sigB* as an “emergency system” that cells utilise under stressful conditions. Consequently, it is a central control mechanism for numerous stress responses. This factor appears capable of regulating the expression of over 150 genes, including those with the *sigB* operon (Hecker et al., 2007; Yeak et al., 2023). The *sigB* operon exhibits similar clusters across various *Bacillus* spp. species, with some differences (e.g., three genes for the *B. anthracis sigB* operon and eight genes for the *B. subtilis sigB* operon) (Fouet et al., 2000). Being integral to the general stress response, it was upregulated from the initial hours of exposure of *B. cereus* and *B. subtilis* to *T. vulgaris* and *O. vulgare* EOs. However, this gene did not appear to be mainly involved in the stress induced by the EO, compared to other genes, especially for *T. vulgaris* treatment. Indeed, *sigB* is widely recognised for its regulatory activity under contrasting stress conditions, primarily due to different types of stress, such as acid or thermal shocks (Hecker et al., 2007).

The genes *plcR* and *nheB*, involved in the virulence of *B. cereus*, were identified in the genome of *B. cereus* strain 11 in a previous study conducted by Purgatorio et al. (2024). *plcR* was particularly significant in *B. cereus* response. According to Gohar et al. (2008), *plcR* regulates genes encoding for proteins that are either secreted or situated at the cell wall, forming the interface between the bacterial cell and its environment, as well as genes related to sporulation, biofilm formation, and the synthesis of extracellular enzymes and toxins. The authors noted that *plcR* transcription was auto-induced just before the onset of the stationary phase, and that the action of spo0A repressed its expression (Gohar et al., 2008). Our results confirmed the initiation of *plcR* transcription after 12 h, with expression increasing up to 48 h, despite the concurrent upregulation of *spo0A*. In *B. cereus, plcR* also serves a crucial role as a virulence regulator in controlling gene transcription for enterotoxins. Hadjilouka et al. (2017) consistently observed the expression of numerous virulence genes following the treatment of *L. monocytogenes* with lemongrass EO. The *nheB* gene was also overexpressed, albeit less than *plcR*, particularly following *T. vulgaris* EO treatment. It regulates extracellular virulence, contributing to the production of non-haemolytic enterotoxin (Hansen and Hendriksen, 2001; Li et al., 2016). Both *nheB* and *pbpF*, a gene associated with peptidoglycan biosynthesis, were significantly upregulated to their maximum levels after 18 h of *T. vulgaris* EO exposure.

The *spo0A* gene is well-known as a sporulation factor that contributes to biofilm formation and regulates the transition phase of growth. It influences the expression of hundreds of genes and can prevent the initiation of DNA replication by binding to the origins of DNA replication. Although this gene is widely studied in the *Bacillus* and *Clostridium* genera, to the best of our knowledge, the behaviour we observed in our study (no expression at 18 h and 24 h under treatment with *Thymus vulgaris*) has never been documented in the literature. Further research is required to confirm this phenomenon in other *B. cereus* strains using different *Thymus vulgaris* EOs; however, this finding likely represents the most significant discovery of our work, at least regarding the use of EO for *B. cereus* control in foods. By controlling sporulation, *spo0A* promotes the transcription of an enzyme that antagonises *sinR*. In this study, *sinR* was expressed in most cases after 18 h of exposure. In *Bacillus thuringiensis, sinR* regulates genes involved in detoxification processes, sugar metabolism, DNA recombination and degradation, peptidoglycan turnover, and energy production. Additionally, this gene represses biofilm formation and is necessary for swimming motility (Fagerlund et al., 2014).

*B. subtilis* strain 58C responded to the stress induced by exposure to *T. vulgaris* EO quite differently from *B. cereus* strain 11. This finding aligns with other authors who reported essential differences between the two species in several regulatory pathways, including those involved in stress response (Fagerlund et al., 2014; Gohar et al., 2008). At the early stages of exposure to *T. vulgaris* EO*, B. subtilis* showed a significant improvement in gene expression, particularly for *pbpF*, *sigB,* and *spo0A*. According to Harms et al. (2024), *sigB*, *spo0A*, and *sinR* are involved in the adaptive response of *B. subtilis* to unfavourable conditions. Our results demonstrate that *sinR* was upregulated but only in the final hours of exposure, indicating that this is not the first gene activated in response to the presence of EOs.

The *codY* gene regulates over two hundred *B. subtilis* genes (Barbieri et al., 2015). During our observation, *codY* and *sinR* remained predominantly down-regulated (*p* < 0.05), and began to be significantly upregulated after 24 h for *O. vulgare* EO and 48 h for *T. vulgaris* EO treatments. These genes regulate the promotion of mobility, flagella expression, and biofilm control (Jin et al., 2021). Some of these functions overlap with those of the *spo0A* gene; however, the latter is more involved in the initiation of sporulation, which appears to play a significant role in stress response (Xu et al., 2017).

*B. subtilis* did not use the same main genetic repair mechanisms as *B. cereus,* favouring the transcription of the gene *pbpF* encoding for a protein family known as penicillin-binding proteins. This observation agrees with Yoshikazu et al. (2009), who described the regulation of cell wall morphogenesis in *B. subtilis* by recruiting PBP1 proteins. The involvement of genes that encode for membrane integrity and peptidoglycan biosynthesis is also reported by Hadjilouka et al. (2017) for *L. monocytogenes*.

The higher sublethal concentration (0.58 mg/mL) generally resulted in more significant gene overexpression than the lower concentration (0.29 mg/mL), likely because the cells, experiencing more severe damage, activate emergency mechanisms more vigorously. For some genes, *O. vulgare* EO resulted in higher expression at 0.29 mg/mL, particularly against *B. cereus*. These differences, along with the fact that for *B. cereus* and *B. subtilis* the two EOs determine slightly different gene expression profiles, are closely related to the composition of EOs. In fact, even substances present in minimal quantities can influence the mechanisms of action against the target cell and how it genetically reacts to the external agent. *T. vulgaris* EO is the thymol chemotype and contains significant amounts of γ-terpinene, *p*-cymene, linalool, and carvacrol. In contrast, *O. vulgare* subsp. *hirtum* EO is the carvacrol chemotype and also includes γ-terpinene, *p*-cymene, and (E)-caryophyllene as its main components. These substances interact with one another and those present at very low concentrations, resulting in effects that could vary significantly between them. Even a phenolic compound present in what might seem like an irrelevant concentration can substantially influence the impact on the cell (D’Amato et al., 2024). Further studies on how the interaction between the different components of antimicrobial substances may affect gene expression would be beneficial.

## 5 Conclusion

The present study provides a first overview of how two of the most common EOs act at the molecular level on *B. cereus* and *B. subtilis strains*. Although further studies are required, the results improve understanding of the effects of EOs on these species, as only a few gene expression studies have focused on these microorganisms and their responses to EOs, especially at sublethal treatments.

The higher sublethal concentration (0.58 mg/mL) was generally more stimulating. Moreover, in most cases, significant upregulation started at 12 h, and continued differently depending on the gene, while at the beginning of the exposure (6 h), most genes were downregulated. These findings also suggest that a better understanding of the molecular mechanisms involved in the repression of these genes could provide a foundation for new research in the field of natural substances used as antimicrobials to replace conventional food preservatives or to substitute or synergise with antibiotics to counteract the rampant phenomenon of antimicrobial resistance. In fact, elucidating the cellular mechanisms implicated in controlling food pathogenic and spoilage microorganisms, along with the concentrations and exposure time at which EOs can exert their activity, would allow for the optimisation of their application to foods. Although some of the genes studied in this work are well-known, further exploration is needed to investigate the complex regulatory pathways triggered by the stress induced by *T. vulgaris*, *O. vulgare* subsp. *hirtum* and other commonly used EOs, considering the significant variability of phenolic compounds and interaction between them, which can influence gene expression profiles.

## Data Availability

The original contributions presented in the study are included in the article, further inquiries can be directed to the corresponding author.

## Footnotes

^1^ https://www.ebi.ac.uk/

^2^ https://blast.ncbi.nlm.nih.gov/

^3^ https://www.gene-quantification.de
